# Microarchitecture of the Augmented Bone Following Sinus Elevation with an Albumin Impregnated Demineralized Freeze-Dried Bone Allograft (BoneAlbumin) versus Anorganic Bovine Bone Mineral: A Randomized Prospective Clinical, Histomorphometric, and Micro-Computed Tomography Study

**DOI:** 10.3390/ma11020202

**Published:** 2018-01-28

**Authors:** Kivovics Márton, Szabó Bence Tamás, Németh Orsolya, Czinkóczky Béla, Dőri Ferenc, Nagy Péter, Dobó-Nagy Csaba, Csönge Lajos, Lacza Zsombor, Mijiritsky Eitan, Szabó György

**Affiliations:** 1 Department of Community Dentistry, Semmelweis University, Szentkirályi utca 40, 1088 Budapest, Hungary; nemeth.orsolya@dent.semmelweis-univ.hu (N.O.); czinkoczky.bela@dent.semmelweis-univ.hu (C.B.); 2 Department of Oral Diagnostics, Semmelweis University, Szentkirályi utca 47, 1088 Budapest, Hungary; szabo.bence_tamas@dent.semmelweis-univ.hu (S.B.T.); dobo-nagy.csaba@dent.semmelweis-univ.hu (D.-N.C.); 3 Department of Periodontology, Semmelweis University, Szentkirályi utca 47, 1088 Budapest, Hungary; dori.ferenc@dent.semmelweis-univ.hu; 4 Department of Pathology and Experimental Cancer Research, Semmelweis University, Üllői út 26, 1085 Budapest, Hungary; nagy@korb1.sote.hu; 5 Petz Aladár County Hospital, West Hungarian Regional Tissue Bank, Vasvári Pál u. 2–4, 9024 Győr, Hungary; tissue@petz.gyor.hu; 6 Institute of Clinical Experimental Research, Semmelweis University, Tűzoltó u. 37–47, 1094 Budapest, Hungary; lacza.zsombor@med.semmelweis-univ.hu; 7 Department of Oral Rehabilitation, Tel Aviv University, Ramat Aviv, Tel Aviv 6997801, Israel; mijiritsky@bezeqint.net; 8 Department of Oro-Maxillofacial Surgery and Stomatology, Semmelweis University, Mária utca 52, 1085 Budapest, Hungary; szabo.gyorgy@dent.semmelweis-univ.hu

**Keywords:** maxillary sinus floor augmentation, bone graft material, xenograft, allograft, x-ray microtomography, histology, histomorphometry

## Abstract

Serum albumin has been identified as an endogenous protein that is integral to early bone regeneration. We hypothesized that albumin addition to allografts may result in better bone remodeling than what can be achieved with anorganic xenografts. Sinus elevations were performed at 32 sites of 18 patients with the lateral window technique. Sites either received filling with an anorganic bovine bone mineral (ABBM, BioOss, Geistlich, CH) or albumin impregnated allograft (BoneAlbumin, OrthoSera, AT). After 6-months patients received dental implants and 16 bone core biopsy samples were obtained from the ABBM filled, and 16 from the BoneAlbumin augmented sites. The biopsies were examined by histomorphometry and µCT. Percentage of the residual graft in the BoneAlbumin group was 0–12.7%, median 5.4% vs. ABBM 6.3–35.9%, median 16.9%, *p* < 0.05. Results of the µCT analysis showed that the microarchitecture of the augmented bone in the BoneAlbumin group resembles that of the native maxilla in morphometric parameters Trabecular Pattern Factor and Connectivity. Our data show that while ABBM successfully integrates into the newly formed bone tissue as persisting particles, BoneAlbumin is underway towards complete remodeling with new bone closely resembling that of the intact maxilla.

## 1. Introduction

Development of the alveolar ridge is dependent on tooth eruption and loss of teeth results in its subsequent resorption. The alveolar process is a tooth dependent structure of the jaws [1,2,3,4]. During the first year after tooth removal, there is a 25% decrease in the volume of the ridge, and its width reduces by 40–60% in the first three years [5,6]. Maxillary sinus pneumatization is characterized by the maxillary sinus extension into the adjacent bone structure. Alveolar pneumatization has been reported in at least 50% of the population [7,8]. Sinus pneumatization and alveolar atrophy frequently cause insufficient bone volume for dental implant placement in the edentulous posterior maxilla. Maxillary sinus floor augmentation with the lateral window technique is a safe and predictable surgical intervention to restore the bone volume of the maxillary premolar and molar area [9]. However, potential complications may include perforation of sinus membrane and bleeding of the alveolar-antral vascular bundle. Preoperative analysis of high-resolution, three-dimension imaging can be considered a method of choice to investigate anatomical variations that may cause complications. New surgical techniques have been reported to prevent intraoperative bleeding during sinus augmentation [10,11,12].

There are reports of successful application of several bone graft materials in sinus augmentation i.e., autologous bone, mineralized freeze-dried bone allograft, demineralized freeze-dried bone allograft, autogenous tooth bone graft material, β-tricalcium phosphate, nanocrystalline hydroxyapatite, bioactive glass, biphasic ceramic bone substitute, anorganic bovine bone mineral (ABBM), and other xenografts of bovine and porcine origin [1,13,14,15,16,17,18,19,20,21,22,23,24,25,26]. Autogenous bone grafts are considered the gold standard due to their osteogenic capacity, however, donor site morbidity and limited availability are disadvantages of their use. It has been reported that patients have significantly more pain and discomfort in the postoperative period when autografts are harvested, therefore, one would rather use an exogenous material in case adequate bone formation can be achieved with it [27].

Allografts overcome the need for a donor region and the limits of harvestable quantity. However, processing of the allograft not only eliminates the host’s immunogenic activity but also the viable osteogenic cells and osteoinductive factors [19,22,28]. One such native bone protein that is integral to regeneration is albumin, which is highly water soluble and washed away during the preservation process. Several studies showed that albumin induces mesenchymal stem cell growth on the surface of freeze-dried bone allograft and albumin impregnation of the bone allograft results in better bone formation even in challenging applications [29,30,31,32,33,34,35,36]. These preclinical and orthopedic studies have shown the biological advantages of BoneAlbumin, raising the possibility that it may also be advantageous in sinus floor augmentation. However, the alveolar ridge of the maxilla poses anatomical challenges to bone grafting and it is not apparently evident if the advantages offered by albumin-impregnation, such as stem cell mobilization and faster remodeling are indeed useful in this location. For example, the maxilla has a fast bone turnover and very high vascularization which may result in accelerated resorption of the graft material before adequate now bone is formed. Further complicating this site is the high anatomical variation of the sinus cavity septa and arteries that require careful preoperative planning [10,11,12].

The aim of this prospective randomized study is to evaluate the remodeling capacity of albumin impregnated freeze-dried bone allograft in sinus floor augmentation by histolomorphometry and μCT analysis and to compare it to anorganic bovine xenograft material.

## 2. Materials and Methods

Patients who were periodontally healthy, more than 18 years of age, and needed implant-supported fixed restoration in the maxillary premolar and molar region were included in this study. The exclusion criteria were as follows: history of uncontrolled systemic diseases known to alter bone metabolism (osteoporosis, diabetes mellitus) or contraindicate oral surgical treatment (tumors and inflammations affecting the maxillary sinuses and the alveolar ridges), patients who were taking or had taken medications known to modify bone metabolism (bisphosphonates, corticosteroids) or had been subjected to irradiation of the maxillofacial region, unwillingness to return for follow-up examinations, smoking, or being pregnant at the time of inclusion. The procedures included in the study were thoroughly explained to the patients, and they signed informed consent forms. The study was approved by the Regional and Institutional Committee of Science and Research Ethics (52158-2/2015/EKU [0425/15]) and The Office of the Chief Medical Officer of The National Public Health and Medical Officer Service (IF-14561-10/2015). The study was conducted in accordance with the Helsinki declaration.

Preoperative planning was performed with the help of cone beam computed tomography (cbCT) and septal sinuses and arterial variations were taken in to consideration when selecting the sites for inclusion in the study [10,11,12]. Patients performed oral rinse with 0.2% chlorhexidine solution for 1 min before surgery. Under local anesthesia a full thickness flap was raised from a midcrestal incision along with two relieving incisions. Lateral window osteotomies were carried out with diamond burs. The Schneiderian membrane was carefully elevated. Bone graft material (2–3 cm^3^) was packed in the defect with light force. Patients were randomly assigned to 2 groups based on the type of bone filler: albumin impregnated allograft (BoneAlbumin, OrthoSera GmbH, Krems an der Donau, Austria) or anorganic bovine bone mineral (ABBM), (Bio-Oss Geistlich Wolhusen, Switzerland). A porcine collagen membrane (Bio Gide, Geistlich GmbH, Wolhusen, Switzerland) was placed over the lateral window and fixed by titanium pins (Titan Pin Set, Ustomed Instruments Ulrich Storz GmbH & Co. KG, Tuttlingen, Germany). The buccal flap was mobilized to allow tension-free primary closure. The margins were stabilized with single interrupted sutures. Antibiotics (1 g amoxicillin-clavulanate twice a day for 5 days, or in case of side effects or known allergy to penicillin, 300 mg clindamycin 4 times a day for 4 days), anti-inflammatory drugs (50 mg diclofenac 3 times a day for 3 days), and chlorhexidine mouthwash (twice a day for 21 days from the first day after surgery) were prescribed. Sutures were removed after 10 days. During the healing period, patients either received temporary prostheses that were in no contact with the surgical area or did not wear them at all. After 6-month healing period, clinical examination and a cbCT was performed followed by surgical re-entry procedure under local anesthesia. A bone core biopsy was taken with a trephine with an external diameter of 3.5 mm and an internal diameter of 2.5 mm (330 205 486 001 025 Hager & Meisinger GmbH, Neuss, Germany) and implants of at least 4.0 mm of diameter were placed into the grafted alveolus nonsubmerged. The bone core biopsy samples were removed, pushed out from the trephine, and placed in sample holders filled with 4% formaldehyde solution in 0.1 M phosphate buffer saline (PBS), pH 7.3, stored at 4 °C. Final prosthetic restorations were cemented after 3 months.

**Figure 1:**
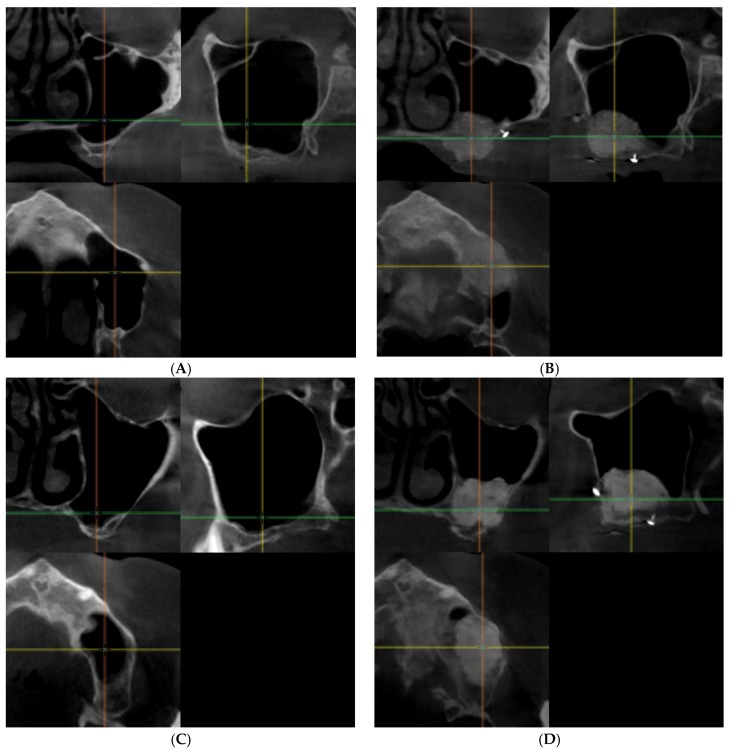
Presents pre- and postoperative cone beam computed tomography (cbCT) images of the augmented sinuses in both study groups. (**A**) preoperative and (**B**) postoperative images of one of the patients of the BoneAlbumin group, (**C**) preoperative and (**D**) postoperative images of one of the patients in the ABBM group. On each image the coronal view is presented on the upper left, the sagittal view is presented on the upper right and the axial view is presented on the lower left window.

Altogether 32 biopsy samples were taken in 18 patients (BoneAlbumin group: 16 implants placed in 12 sinus lifts in 9 patients (3 male), age 54.7 ± 6.5 years, ABBM group: 16 implants placed in 11 sinus lifts in 9 patients (2 male), age 51.1 ± 15.8 years). Figure 1 presents pre- and postoperative cbCT images of the augmented sinuses.

Bone core biopsy material was fixed in 10% buffered formaldehyde solution. Following decalcination and dehydration, the biopsy material was embedded in paraffin and 20 µm sections were prepared. The sections were stained with routine haematoxylin-eosin stain. Sections were evaluated under a light microscope in magnification 40×–400×.

Histomorphometric measurements were completed on sections with Panoramic Viewer 1.15 (3DHISTECH Ltd., Budapest, Hungary) using a combination of Adobe PhotoShop (Adobe System Inc., San Jose, CA, USA) and ImageJ, the public domain NIH Image program (US National Institutes of Health’ http://rsb.info.nih.gov/nih-image/) at 150× magnification. Two slides of the augmented areas in each bone core biopsy sample were evaluated to record area percentage of newly formed bone, residual particles of the bone graft material, and the bone marrow space according to published protocols [37,38]. Histomorphometric data consisted of the area percentage of newly formed bone, residual particles of the graft material and bone marrow were identified in each section by an investigator blinded to the treatment group.

The bone core biopsy samples were scanned using a microcomputed tomography (μCT) scanner (Skyscan 1172 X-ray microtomograph, Bruker µCT, Kontich, Belgium). Bone core biopsy samples were scanned in a 4% buffered formaldehyde solution. Scanning was carried out at a resolution of 5.9 µm (70 kV, 124 µA). A 0.5 mm aluminium filter was used to reduce image noise. Average scan duration was 25 min. After the acquisition, raw images were reconstructed by using NRecon software (v.1.7.1.6., Bruker µCT, Kontich, Belgium). Ring artefact correction was 10, and the beam hardening correction was 61%. The morphometric variables relevant to our study calculated by CTAn software (v.1.17.7.2, Bruker µCT, Kontich, Belgium) and their definitions are listed in Table 1 [39,40]. On the reconstructed images of each sample the demarcation plane of the host and the augmented area was identified and the complete available tissue (maxilla or augmented bone, respectively) were selected as regions of interest (ROIs) for quantitative analysis. Since the length of the biopsy depended on the thickness of the ridge and the graft/host ratio was variable among the patients the volumes of the selected ROIs were not uniform throughout the study. Therefore, we opted for measuring the complete available sample and analyze the data using volume-independent metrics.

Micromorphometric data was collected by evaluation of the augmented bone of the test or control group. Additional micromorphometric data was recorded by analyzing the native bone of the alveolar ridge within the bone core biopsy samples of both test and control group. The microarchitectural parameters of the augmented bone of the BoneAlbumin group and the ABBM group; the augmented bone of the BoneAlbumin group and the native bone of the alveolar ridge; the augmented bone of the ABBM group and the native bone of the alveolar ridge were compared.

Percentage values of each bone core biopsy sample were used to calculate descriptive statistics for the hisomorphometrical and quantitative µCT analysis. The results were analyzed statistically using the IBM SPSS Statistics 23 data analysis software program (IBM Corporation, New York, NY, USA). The Kruskal Wallis One-way ANOVA test was used to compare two sets of data for the statistical analysis of the histomorphometric results. One-way ANOVA test was used to compare three sets of data for the statistical analysis of the micromorphometric results of the μCT. Values of *p* < 0.05 were considered statistically significant.

## 3. Results

### 3.1. Histology and Histomorphometry

At the time of re-entry, care was taken to preserve the anatomical orientation of the bone core biopsies. In both study groups the bone substitute material integrated in the augmented areas with no sign of inflammation or foreign body reaction. Altogether 1 out of 32 implants failed at 1 year follow up in the entire study (BoneAlbumin group); the cause of failure was the lack of osseointegration which was independent of the grafting procedure.

**Figure 2:**
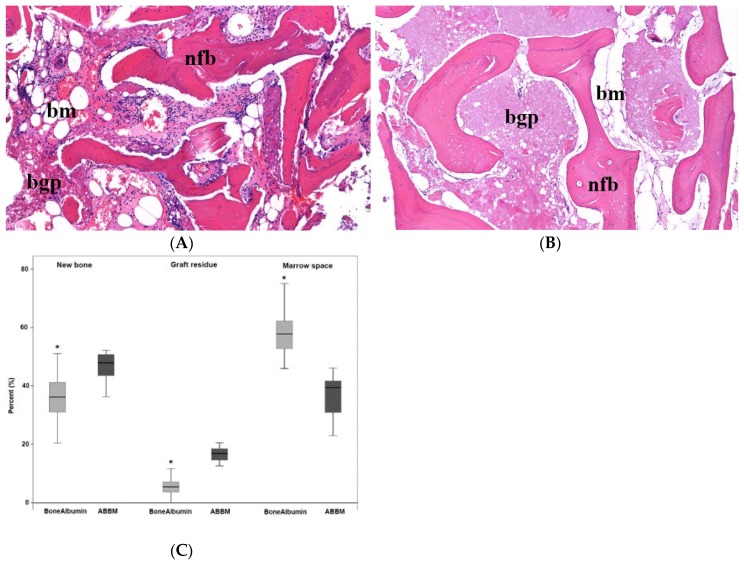
Histological assessment of the graft and the newly formed bone tissue. (**A**) A representative histological section with Hematoxylin-Eosin staining of a bone core biopsy sample harvested from the area augmented using BoneAlbumin. New trabecular bone formation (newly formed bone, nfb) is evident and the intertrabecular space is filled with bone marrow (bm). Only limited acellular graft residues (bone graft particles, bgp) are seen. (**B**) A corresponding ABBM grafted section. Trabecular new bone (nfb) is present in a similar manner; however, the marrow (bm) is partially filled with the residues of the acellular graft material (bgp). Panel (**C**) shows the histomorphometry data of the stained sections. Graft residues in the BoneAlbumin group are significantly less than those grafted with ABBM, at the expense of marrow space. Data are presented as median–quartiles–ranges, * represents *p* < 0.05 with Kruskal Wallis One-way ANOVA test between the two experimental groups, respectively.

Histological analysis of the bone core biopsy samples obtained from the augmented sites of the BoneAlbumin group revealed that particles of the albumin impregnated allograft underwent considerable resorption and remodeling (Figure 2). Residual particles of the bone graft material were surrounded by newly formed trabecular bone and marrow spaces. In 3 bone core biopsy samples the allograft underwent complete remodeling with no residual bone graft particles present (Figure 2). In contrast, histological analysis of the bone core biopsy samples obtained from the augmented sites of the ABBM group revealed that the particles of the xenograft integrated in the augmented sinuses. Particles of the xenograft intermixed with newly formed bone and marrow spaces surrounding the particles (Figure 2).

### 3.2. μCT Results

#### 3.2.1. Qualitative Analysis of the μCT Images

**Figure 3:**
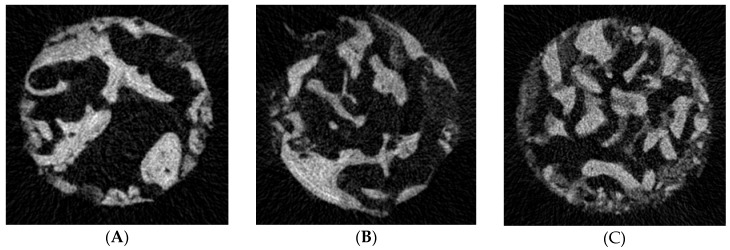

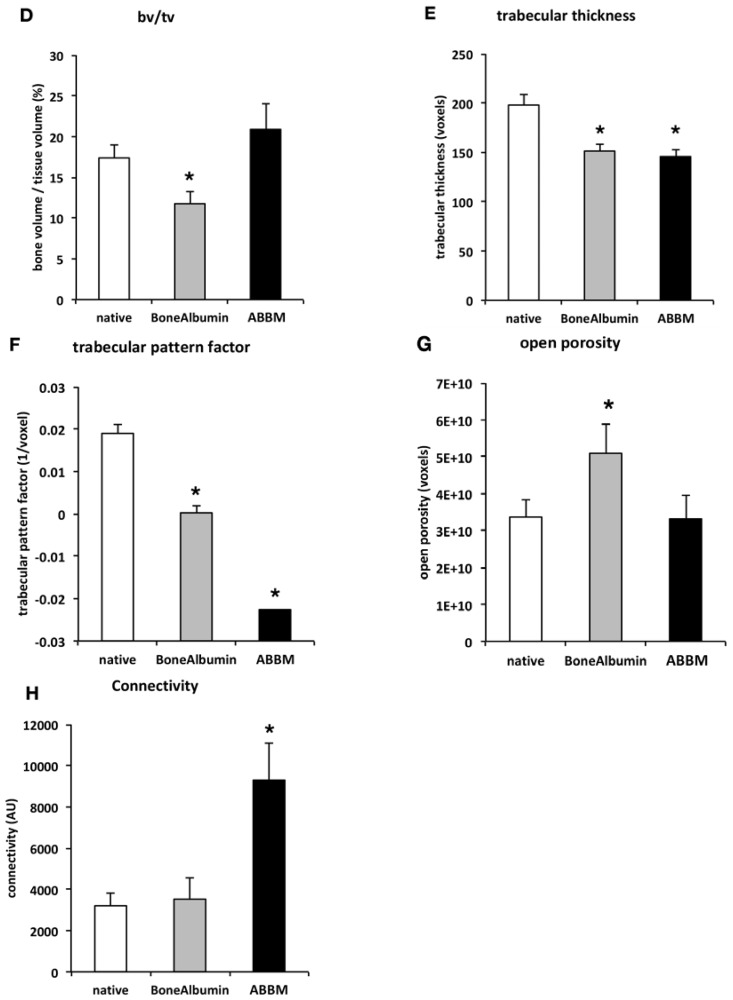
Panels (**A**–**C**) show representative µCT images of the bone core biopsy samples at the level of the residual native bone of the alveolar ridge (**A**), the level of the augmented bone grafted using BoneAlbumin (**B**) and ABBM (**C**). Bone volume fraction (BV/TV) is still somewhat lower the BoneAlbumin group, while in the ABBM group it is comparable to that of native bone—note that what is calculated as ‘bone’ in this metric contains the unresorbed graft ceramic (**D**). Trabecular thickness of the BoneAlbumin group has not yet reached the level of that of the native bone (**E**). Trabecular pattern factor (**F**) which describe bone geometry is closer to native bone in the BoneAlbumin group than in the ABBM group. Open porosity is still somewhat higher than native in the BoneAlbumin group (**G**), while connectivity has reached the native level (**H**). Please note that in these parameters the bone value is a surrogate number for the newly formed bone and the residual graft. * signals *p* < 0.05 versus native bone.

Qualitative analysis of the μCT images of the bone core biopsy samples obtained from the augmented sinuses of both study groups showed marked difference in the appearance of the residual native bone of the alveolar ridge and the augmented bone. Native bone of the alveolar ridge contained thick trabeculae with wide marrow spaces in the biopsy specimens of both study groups. In the augmented bone of the BoneAlbumin group particles of the albumin impregnated allograft and the newly formed bone of the augmented area could not be differentiated based on radiolucency. The augmented bone of the BoneAlbumin group consisted of trabeculae—thinner than that of the native bone—in a complex structure with abundant nodes. In the augmented bone of the ABBM group particles of the xenograft were characterized by greater radiodensity than the newly formed bone. Particles of the xenograft were surrounded and connected by thin trabeculae with a complex structure. The BoneAlbumin augmented tissue resembled a similar structure than that of the native bone. Figure 3 presents representative µCT images of the bone core biopsy samples.

#### 3.2.2. Quantitative Analysis of the μCT Images, Bone Microarchitecture Analysis

Quantitative analysis of the µCT confirmed the observations of the qualitative analysis. Native bone of the alveolar ridge and augmented areas were differentiated based on their appearance in both study groups. Morphometric data of the augmented areas of the BoneAlbumin group, the ABBM group and of the native bone of the alveolar ridge were compared. Open porosity was higher in the BoneAlbumin group, probably due to the denser marrow of the resorbing graft, while bone volume fraction (BV/TV) is higher in the ABBM group due to the persisting high density graft particles. The thickness of the newly formed trabeculae has not yet reached that of the native bone. Trabecular pattern factor and Connectivity, metrics which reflect the microstructure of bone is approaching values of the native bone, more in the BoneAlbumin group than in the ABBM group. The values of the morphometric parameters that showed significant difference between the three sets of data and the results of the statistical analysis are presented in Figure 3.

## 4. Discussion

In the present study we observed that a serum albumin enhanced bone allograft creates a close-to-normal bone architecture. The anorganic xenograft persists and new bone is only growing into the spaces around the particles of the graft material. Both histomorphometry and µCT analysis confirmed that the new bone formed parallel with the resorption of BoneAlbumin and had a structure closely resembling that of the native maxilla.

In several earlier studies µCT was employed to evaluate the microarchitecture of bone fillers following two stage maxillary sinus floor augmetation. In these studies, the bone substitute materials used were autogenous bone, anorganic bovine bone mineral (ABBM), synthetic β-tricalcium phosphate (β-TCP), synthetic biphasic calcium phosphate, and porous titanium granules [41,42,43,44,45,46,47,48,49,50,51].

Anorganic bovine bone mineral is a poorly soluble calcium phosphate ceramic that is the result of high-temperature treatment during processing. Clinical experience suggests that ABBM particles integrate well in newly formed bone but undergo little resorption [52,53,54,55]. In contrast, freeze-dried bone allografts are not subjected to elevated temperatures and the chemical composition of the inorganic bone material is largely preserved. Allografts are typically preserved by lyophilization which maintains the structure of the native bone and osseous apatite content as well as some of the structure proteins and growth factors such as Bone Morphogenic Proteins (BMPs), however the native albumin content of bone is washed away in standard allograft processing [56,57]. Limited growth of Ca^2+^ concentration in ABBM augmented sinuses and increased remodeling in BoneAlbumin augmented sites explain that the percentage of residual bone graft material and percentage of newly formed bone was significantly lower in the biopsy samples obtained from sinuses grafted with BoneAlbumin than in those obtained from sinuses grafted with ABBM. In the augmented bone of the ABBM group particles of the xenograft were characterized by greater radiodensity than the newly formed bone unlike in the augmented bone of the BoneAlbumin group where residual allograft particles were indistinguishable from newly formed bone. This phenomenon can also be explained by the different manufacturing procedures of the two initially similar raw materials, namely bovine or human bone. Bovine bone is subjected to high temperatures during production, which enhances crystallinity resulting in a more radio dense material while BoneAlbumin is manufactured by the Urist-protocol which includes partial demineralization of the graft, producing a bone substitute with radiodensity similar to that of native bone [57,58].

The findings of the µCT analysis were consistent with those of the histological and histomorphometric analysis. The microarchitecture of the ABBM augmented bone consisted of residual bone graft particles in close contact with the newly formed bone surrounding it forming a dense structure with thin trabecules and plate like marrow spaces, which is well described in the literature [42,45]. Although the endpoint of the current study was 6 months, the persistence of mineralized xenograft particles is documented for much longer, even decades, indicating that this is the final stage of remodeling and some xenograft particles will remain in the human tissue until the lifetime of the patient [53,54,55,59]. The dense structure showed little orientation in any direction. However, the microarchitecture of the BoneAlbumin augmented bone consisted of extensively resorbed residual bone graft particles surrounded by newly formed bone forming thin, plate and cylinder like trabecules with abundant marrow spaces. This observation is very similar what was previously described in the patella [34].

The Higher Open porosity of the BoneAlbumin group compared to the native bone and the ABBM group is due to the wide, interconnected marrow spaces of the remodeling bone. The Bone volume fraction in the ABBM group is comparable to that of the native bone and higher than that of the BoneAlbumin group. The Trabecular thickness of the BoneAlbumin group and the ABBM group is significantly lower than that of the native bone. However, in these metrics, newly formed bone and residual graft particles are calculated as bone. This means that, as the remodeling of the BoneAlbumin is underway, these parameters are expected to reach the values of the native bone. The unresorbed graft particles of ABBM, however, have integrated into the bone after 6 months and their remodeling is not expected. In three cases of the current study there was no sign of the remaining graft material in the BoneAlbumin group, indicating that even at 6 months’ time the remodeling can be complete. The microarchitecture of the bone in the BoneAlbumin group resembled more to that of the native bone of the alveolar ridge, than that of the ABBM augmented ones when Tb.Pf and Conn. values of bone microarchitecture are observed.

The same is observed with trabecular pattern factor (Tb.Pf), which describes the fractal dimensions of the trabecular organization indicating the organic growth of the bone material. Interestingly, trabecular pattern factor was also shown to be significantly better in an animal study where native bone graft was compared to albumin-coated bone grafts, hence this effect is probably attributable to the albumin addition only [31].

One useful and fast algorithm for calculating the Euler connectivity in 3D is the “Conneulor”. It measures what might be called redundant connectivity, the degree to which parts of the object are multiply connected [40]. The connectivity of the augmented bone of the ABBM group was significantly higher than that of the augmented bone of the BoneAlbumin group and that of the native bone of the alveolar ridge. The connectivity of the augmented bone of the BoneAlbumin group and that of the native bone of the alveolar ridge showed no statistically significant difference. The dense structure of residual particles of the non-resorbable material (ABBM) surrounded by thin trabecules of newly formed bone is responsible for higher connectivity values.

In addition to being less processed and obviously more ‘human’ than ABBM, BoneAlbumin is saturated with serum albumin in a lyophilized form. Empirical observations in any stem cell lab proves that serum albumin in up to 10% concentrations must be a standard constituent of cell culture media, otherwise the bone marrow derived mesenchymal stem cells (BMSCs) just do not grow. In the native environment of the bone and marrow niche, bone cells generate albumin and secrete it into the extracellular space as the first protein in case of bone injury [36]. It has also been shown that albumin is physiologically present in bone tissue, therefore, replenishing the albumin content of bone allografts is required if one tries to build a graft as close to the native tissue as possible [30,31,32,33,34,35]. Several earlier studies in both animals and humans have shown that albumin augmentation results in better bone formation, however, this is the first study that shows its beneficial effects in the challenging environment of the maxilla [30,32,33,34].

The justification of using bone fillers that never fully resorb comes from the clinical concern of premature resorption. While this is not an issue in most orthopedic indications, the high metabolic turnover and exposed anatomy of the alveolar ridges make these locations especially prone to the resorption of a bone filler [52,57,60]. Supplementation of the bone marrow niche with albumin kick-started a bone regeneration process that resulted in new bone closely resembling that of the native maxilla. The bone graft can thus perform its original purpose of directing new tissue formation while the graft material disappears completely, leaving only the patient’s own new bone in its place. As the patient population for sinus lift is expected to live several decades with their new bone, it is especially important that it is native bone which remodels and ages naturally during their lifetime. Limitations of the current study are the relatively short investigational timescale and the microstructural nature of the outcome measures, that are sometimes hard to understand from a clinical point of view. Percentage values of each bone core biopsy sample were used to calculate descriptive statistics for the hisomorphometrical and quantitative µCT analysis. In some cases, more than one bone core biopsy specimens were harvested from the same patient which might be a limitation of this study. The use of ABBM is evidently adequate as a bone filler in the maxilla despite the persistent graft particles and the disturbed histological picture. One would even argue that the remaining graft residue is beneficial to hold the shape and may efficiently support the implant. The timeline of the current investigation is limited to 6 months, while the true clinical benefits of a fully remodeled graft may only be important years or decades later when differences of ageing between a native bone tissue and bone with residual grafts become evident. However, this question is currently only speculative, and a register-type study may answer it when enough BoneAlbumin grafts are implanted and time elapsed.

## 5. Conclusions

In our study we successfully utilized maxillary sinus floor augmentation with a lateral window technique with anorganic bovine bone mineral or albumin impregnated allograft to augment the posterior maxilla prior to implantation. Data from the histological, histomorphometrical and μCT analysis concluded that ABBM integrates with new bone while BoneAlbumin mainly undergoes remodeling.

## Figures and Tables

**Table 1:** The morphometric variables relevant to our study calculated by the CTAn software (according to Bouxsein, M.L., et al. (2010), Gunderson, H.J.G., et al. (1993) and the manual Bruker MicroCT Morphometric parameters measured by CT-analyzer software 1.15.4.0 by Bruker microCT).

Abbreviation	Variable	Description	Standard Unit
BV/TV	Bone volume fraction	Relative volume of calcified tissue in the selected volume of interest (VOI).	%
Tb.Th	Trabecular thickness	Mean thickness of trabeculae, assessed using direct 3D methods.	mm
Tb.Pf	Trabecular bone pattern factor	This is an index of connectivity of trabecular bone; it calculates an index of relative convexity or concavity of the total bone surface, on the principle that concavity indicates connectivity (and the presence of “nodes”), and convexity indicates isolated disconnected structures (struts).	1/mm
Po(op)	Open porosity (percent)	Percent open porosity is the volume of open pores as a percent of the total VOI volume.	%
Conn.	Connectivity	One useful and fast algorithm for calculating the Euler connectivity in 3D is the “Conneulor”. It measures what might be called “redundant connectivity”, the degree to which parts of the object are multiply connected. It is a measure of how many connections in a structure can be severed before the structure falls into two separate pieces.	none
